# Comparative analysis and trends in liver transplant hospitalizations with *Clostridium difficile* infections: A 10‐year national cross‐sectional study

**DOI:** 10.1111/tid.13985

**Published:** 2022-11-14

**Authors:** Hassam Ali, Pratik Patel, Rahul Pamarthy, Karina Fatakhova, Nicole Leigh Bolick, Sanjaya Kumar Satapathy

**Affiliations:** ^1^ Department of Internal Medicine East Carolina University/Vidant Medical Center Greenville North Carolina USA; ^2^ Department of Gastroenterology Mather Hospital/Hofstra University School of Medicine Port Jefferson New York USA; ^3^ Department of Dermatology University of New Mexico University Albuquerque New Mexico USA; ^4^ Department of Hepatology Northshore University Hospital/Hofstra University School of Medicine Manhasset New York USA

**Keywords:** clostridium infections, graft rejection, liver transplantation, mortality, risk factors

## Abstract

**Goals and background:**

*Clostridium difficile* infection (CDI) is the leading cause of antibiotic‐associated diarrhea in the United States. We aimed to determine comparative trends in inpatient outcomes of liver transplant (LT) patients based on CDI during hospitalizations.

**Methods:**

The national inpatient sample database was used to conduct the present retrospective study regarding CDI among the LT hospitalizations from 2009 to 2019. Primary outcomes included 10‐year comparative trends of the length of stay (LOS) and mean inpatient charges (MIC). Secondary outcomes included comparative mortality and LT rejection trends.

**Results:**

There was a 14.05% decrease in CDI in LT hospitalizations over the study period (*p* = .05). The trend in LOS did not significantly vary (*p* = .9). MIC increased significantly over the last decade in LT hospitalizations with CDI (*p* < .001). LT hospitalizations of autoimmune etiology compared against non‐autoimmune did not increase association with CDI, adjusted odds ratio (aOR) 0.97 (95% confidence interval [CI] 0.75–1.26, *p* = .87). CDI was associated with increased mortality in LT hospitalizations, aOR 1.84 (95% CI 1.52–2.24, *p* < .001). In‐hospital mortality for LT hospitalizations with CDI decreased by 7.75% over the study period (*p* = .3). CDI increased transplant rejections, aOR 1.3 (95% CI 1.08–1.65, *p* < .001). There was a declining trend in transplant rejection for LT hospitalization with CDI from 5% to 3% over the study period (*p* = .0048).

**Conclusion:**

CDI prevalence does not increase based on autoimmune LT etiology. It increases mortality in LT hospitalizations; however, trend for mortality and transplant rejections has been declining over the last decade.

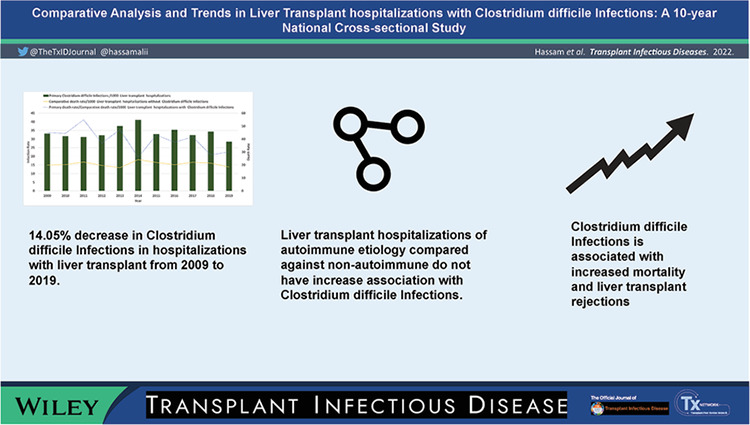

## 1 INTRODUCTION

*Clostridium difficile* infection (CDI) is the leading cause of antibiotic‐associated diarrhea in the United States.^1^ Is it estimated that 50% of antibiotic prescriptions are inappropriate,^2^ which has led to the advent of widespread hospital–based antimicrobial stewardship programs (ASPs). A meta‐analysis revealed a risk ratio for the development of CDI of 0.48 after the implementation of ASPs.^3^ There is a decrease in the estimated burden of CDI in the United States by 24% from 2011 to 2017, which is at least partly explained by the increased presence of ASPs. The decreased overall burden is secondary to a decline in healthcare‐associated CDI (HCA‐CDI), but no change in community‐associated CDI (CA‐CDI), which represents 50% of CDIs.^4^ The recent estimated annual attributable healthcare cost of CDI is $6.3 billion with an estimated 2.4 million days of inpatient stay.^5^ Liver transplant (LT) recipients are at a particularly high risk of developing infections, especially within the first year post‐LT. Nosocomial and opportunistic infections can be seen within the first year post‐LT. A recent study reports that 55% of these patients may experience an infection within the first year post‐LT.^6^ The risk of infection can be explained by frequent hospitalizations, immunosuppressant/antibiotics use, proton pump inhibitors, and comorbidities.^7^ Rate of CDI in solid‐organ transplant recipients has been described to be as high as 40%.^8^ There are no studies evaluating the risk of CDI in LT in addition to trends in mortality and admissions over a 10‐year period.^9^, ^10^, ^11^, ^12^, ^13^, ^14^, ^15^, ^16^ Preventing post‐LT complications such as CDI remains vital for improving post‐LT mortality.^17^ Despite the reported decrease in CDI burden overall, it is unclear if this trend holds true for CDI in the LT population.^4^ Given widespread implementation of ASPs, newer therapies for CDI, and improvement in hygiene, it is possible that a similar decline in CDI is seen in post‐LT patients. We aim to analyze trends in admissions, economic impact, and mortality of CDI in the LT population from 2009 to 2019.

## 2 METHODS

### 2.1 Design and data source

The national inpatient sample (NIS) database was used to conduct the present retrospective study regarding CDI among the LT population from 2009 to 2019.^18^ The etiology of LT was classified as autoimmune including primary biliary cholangitis (PBC), primary sclerosing cholangitis (PSC) and autoimmune hepatitis (AIH), and non‐autoimmune (all others). The NIS is the largest publicly available all‐payer inpatient database in the United States. It has a 20% stratified sample of all US community hospital discharges. Inclusion criteria included patients with a primary diagnosis of an LT as defined by their International Classification of Diseases (ICD) 9 (before September 2015) and 10 (after October 2015) coding systems. The exact codes utilized in this study for each variable can be found in Table S1. All patients below the age of 18 were excluded. Additional information on NIS's design and sampling methods is available at https://www.hcup‐us.ahrq.gov.

### 2.2 Outcome measures

Primary outcomes of interest included 10‐year trends of demographic characteristics, comparative length of stay (LOS), and mean inpatient charges (MIC) (adjusted to 2019 dollars) among the LT hospitalizations with or without CDI. Secondary outcomes included comparative and mortality trends among the LT hospitalizations with or without CDI and the rate of LT rejection.

### 2.3 Statistical analysis

Analyses were performed using statistical software for data science (STATA) version 16.0 software (StataCorp LLC, Station, TX, USA). Our analysis had .05 as the threshold for statistical significance, and all *p*‐values were two sided. Patient characteristics were compared using a Chi‐squared test for categorical variables and an independent‐samples *t*‐test for continuous variables. Categorical variables were presented as frequency (*N*) and percentage (%), and continuous variables were reported as mean with standard deviation (SE) as appropriate. As per the Agency for Healthcare Research and Quality, the total number of hospitalizations/year was weighted to provide a nationwide estimate.^19^ Hierarchical multivariate linear regression analysis was conducted to adjust the patient or hospital‐level factors and common risks for CDIs like inflammatory bowel disease (IBD) as in prior studies.^20^, ^21^, ^22^ We utilized the adjusted Wald test to compare slopes of time‐based linear regression outcomes and margin plots command in STATA to generate figures.^23^, ^24^, ^25^ Only variables, associated with the outcome of interest on univariate regression analysis at *p* < .2 or known potential confounders despite a *p*‐value indicating no significance, were used in the multivariate regression. Logistic regression outcomes were reported as adjusted odd ratios (aOR) with 95% confidence intervals (CI) and *p*‐value.

### 2.4 Ethical consideration

NIS contains de‐identified third‐party data. Therefore, it was deemed exempt from the institutional review board. NIS also does not include patient identifiers; therefore, patient consent was waived.

## 3 RESULTS

There were 15 457 (3.36%) LT hospitalization cases with CDI for the study period. There was male (56%) and White race (77%) predominance (Table S2). The mean age was not significantly different among patients with or without CDI (*p* = .28). Among these hospitalizations, patients with CDI had a higher Charlson Comorbidity Index score of ≥3 than those without infection (61% vs. 59%, *p* = .0039). Patients with CDI had a higher prevalence of IBD than those without infection (6% vs. 3%, *p* < .001).

**FIGURE 1:**
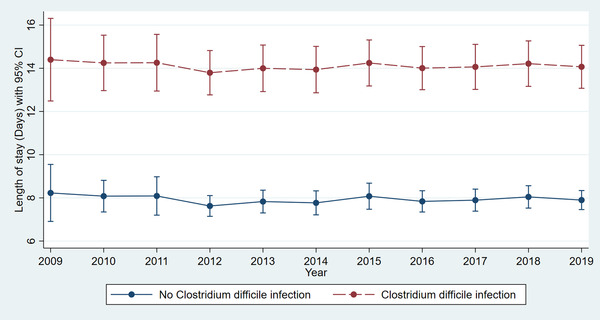
Comparative trend analysis of hospital length of stay (LOS) among liver transplant patients with or without *Clostridium difficile* infection

**FIGURE 2:**
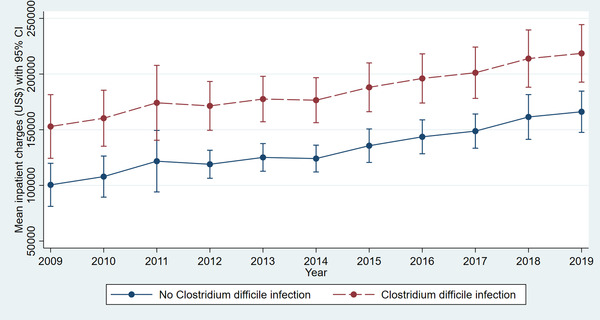
Comparative trend analysis of hospital mean inpatient charges (MIC) among liver transplant patients with or without *Clostridium difficile* infection

Adjusted linear regression revealed a significantly higher LOS in LT hospitalizations with CDI compared to those without infection (14.52 vs. 7.56 days, *p* < .001) for the study period (Table S2). The trend in LOS over the study period did not vary significantly for LT hospitalizations with CDI (Figure 1) (*p* = .9). Adjusted linear regression revealed a significantly higher MIC in LT hospitalizations with CDI compared to those without infection ($200 419 vs. $123 443, *p* < .001) for the study period (Table S2). The MIC increased from $150 517 in 2009 to $216 179 in 2019 for LT hospitalizations with CDI (Figure 2) (*p* < .001).

**FIGURE 3:**
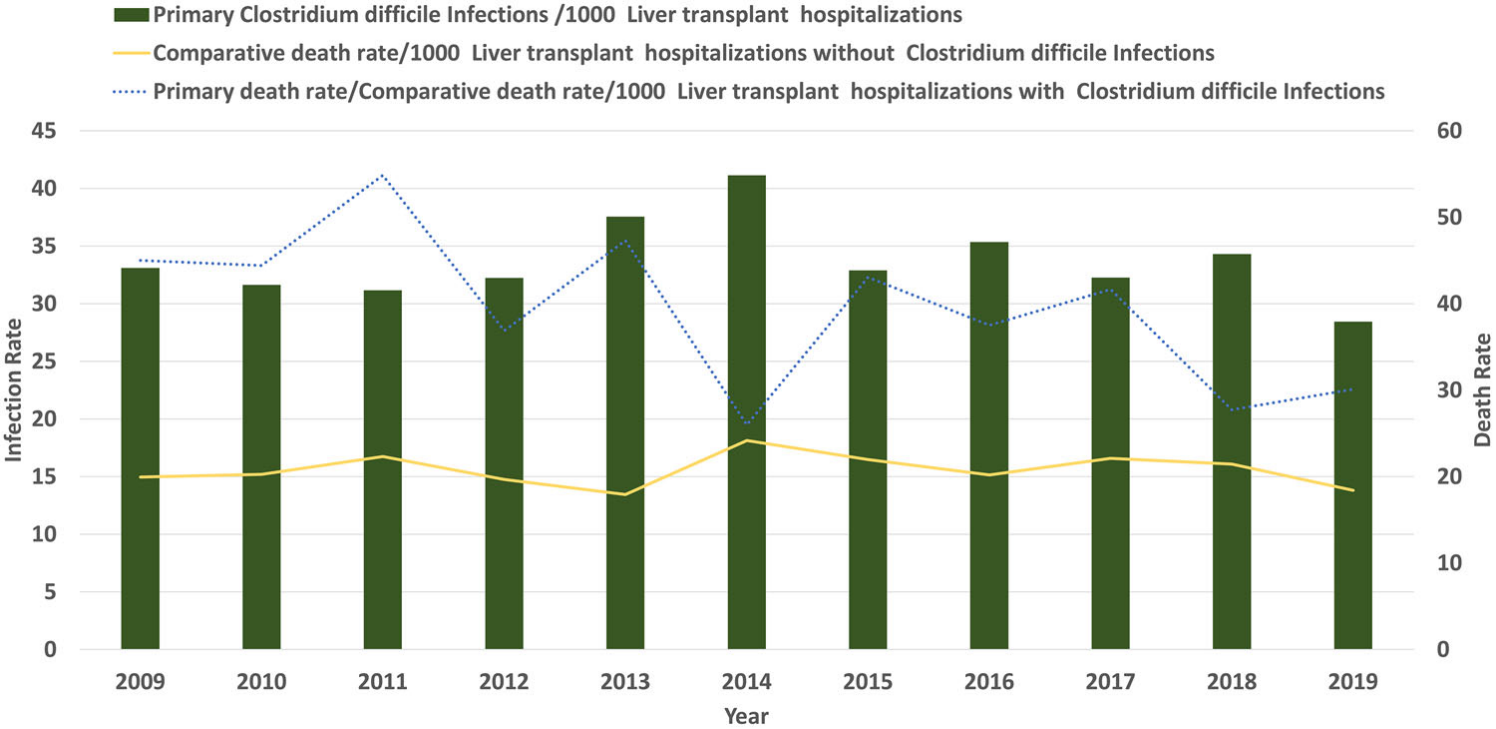
Rate of occurrence of *Clostridium difficile* infection (CDI) and associated mortality compared with mortality for non‐CDIs for liver transplant (LT) patients. Bars show the infection rate per 1000 total LT hospitalizations. The dotted line shows the mortality rate in CDIs per 1000 LT hospitalizations. The continuous line shows the comparative mortality rate in non‐CDI per 1000 LT hospitalizations.

**FIGURE 4:**
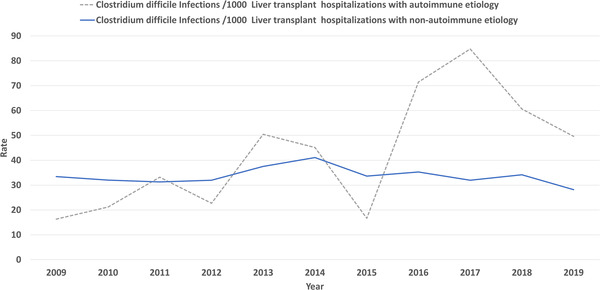
Rate of occurrence of *Clostridium difficile* infection (CDI) based on etiology for liver transplant (LT). The continuous line shows the rate of CDIs per 1000 LT hospitalizations without autoimmune etiology. The dotted line shows the comparative rate of *C. difficile* per 1000 LT hospitalizations with autoimmune etiology.

We report a 14.05% decrease in CDI in LT hospitalizations with the rate decreasing from 33.11 per 1000 LT hospitalizations in 2009 to 28.455 per 1000 in 2019 nearing significance (Figure 3) (*p* = .050). Based on etiology (autoimmune vs. non‐autoimmune), there was no significant difference in the prevalence of CDI in LT patients (3% each, *p* = .71) (Table S2). LT hospitalizations of autoimmune etiology compared against non‐autoimmune etiology did not have a significant increase in association with CDI, aOR 0.97 (95% CI 0.75–1.26, *p* = .87). Patients with autoimmune liver disease had a rising rate of CDI from 16.3 in 2009 to 49.59 per 1000 LT hospitalizations in 2019, without statistical significance (*p* = .34). Patients with non‐autoimmune liver disease had a declining rate of CDI from 33.4 in 2009 to 28.17 per 1000 LT hospitalizations in 2019, nearing significance (*p* = .07) (Figure 4). Overall mortality for all hospitalizations for the study period was significantly higher in LT hospitalizations with CDI versus no infection (4% vs. 2%, *p* < .001) (Table S2). CDI was associated with increased mortality in LT hospitalizations, aOR 1.84 (95% CI 1.52–2.24, *p* < .001). On trend analysis, in‐hospital mortality for LT hospitalizations without CDI decreased by 7.75% (from 19.966 deaths per 1000 LT hospitalizations in 2009 to 18.417 deaths per 1000 LT hospitalizations in 2019) without statistical significance (*p* = .3). In‐hospital mortality for LT hospitalizations with CDI decreased by 7.75% (from 45 deaths per 1000 LT hospitalizations in 2009 to 30.1 deaths per 1000 LT hospitalizations in 2019) without statistical significance (*p* = .3).

Hospitalizations with CDI had a higher frequency of LT rejection (3% vs. 2%) than those without infection. On adjusted regression analysis, CDI was associated with increased transplant rejections, aOR 1.3 (95% CI 1.08–1.65, *p* < .001). There was a declining trend in transplant rejection for LT hospitalization with CDI from 62 (5%) in 2009 to 30 (2%) in 2019 (*p* = .0048). Ten‐year trends for outcomes of interest are further described in Table S3.

## 4 DISCUSSION

*C. difficile* is a gram‐positive bacillus that is transmitted via fecal–oral route. Antibiotic use is the most significant overall risk factor for the development of CDI. The most common antibiotics implicated in the development of CDI are ampicillin, amoxicillin, cephalosporins, clindamycin, and fluoroquinolones.^26^ Many of these antibiotics, including trimethoprim–sulfamethoxazole, are commonly used in the post‐LT period, whether for prophylaxis or for treatment of other infections. CDI presents a major strain on patient health and a major burden on the healthcare industry. These difficulties are exacerbated in the immunocompromised, for example, LT recipients. LT recipients are at a higher risk for more severe CDI and thus more complicated hospitalizations. The results from our study confirmed these beliefs as CDI significantly increased the LOS and MIC for hospitalized LT recipients.

Our results revealed a decrease in the rates of CDI in LT patients from 2009 to 2019 nearing significance (*p* = .05). It can be hypothesized that lower antibiotic use due to general hospital‐based ASPs has a role in this, especially in recent years, as the recent decline in CDI burden is secondary to a decline in HCA CDI.^2^, ^4^ Although HCA‐CDI rates continue to decline, CA‐CDI has remained stable.^4^ In contrast to HCA‐CDI, CA‐CDI is typically seen in the younger population (age < 65).^27^, ^28^ The younger age group represents the majority of LT recipients as the Organ Procurement and Transplantation Network/Scientific Registry of Transplant Recipients data from 2020 revealed that 71% of LT recipients were younger than 65.^29^ This highlights the importance of educating LT recipients of the risks of CDI and the measures that can be used to prevent the transmission of CDI, particularly in out‐of‐hospital settings.

LOS for LT patients with CDI remained stable from 2009 to 2019 despite the introduction of fidaxomicin, bezlotoxumab, and the increased use of fecal microbiota transplant (FMT). Fidaxomicin is a macrocyclic lactone antibiotic that inhibits RNA polymerase of *C. difficile*. It was approved by the Food and Drug Administration (FDA) in 2011 and is now a mainstay in the management guidelines for CDI. Bezlotoxumab is a fully human monoclonal antibody that binds to *C. difficile* toxin B, which was shown to be effective in managing recurrent CDI.^30^ Bezlotoxumab was FDA approved in 2016. Although fidaxomicin is widely used amongst all patient populations with CDI, there is limited data on the use of bezlotoxumab and FMT in solid‐organ transplant recipients. Johnson et al. were able to show in 94 patients that bezlotoxumab significantly reduced recurrent CDI among solid‐organ transplant recipients (OR 0.28 [95% CI, 0.08–0.91]).^31^ Cheng et al. performed a retrospective study that revealed a good safety profile and an overall CDI cure rate of 91.3% with FMT among 94 patients with solid‐organ transplants.^32^ Increased use of bezlotoxumab and FMT for appropriate LT patients with CDI may help improve overall outcomes. More research is required to assess the safety profile and efficacy of bezlotoxumab and FMT in the post‐LT population.

Mortality for LT recipients in our cohort was twice as high if they were hospitalized with CDI. Additionally, LT recipients with CDI experienced a slight increase in transplant rejection. A single‐center retrospective cohort study by Mittal et al. involving 970 LT recipients also revealed an increased mortality rate in LT recipients with CDI versus no CDI (35% vs. 26%, *p* = .003).^12^ Increased mortality with CDI is also replicated in the non‐LT population. Olsen et al. were able to show a significant increase in mortality (OR 1.77; 95% CI, 1.74–1.81) for overall admissions with CDI compared to control.^33^ The immunosuppressed state of LT recipients likely explains the increased susceptibility to severe infections and death. The increased risk of rejection and graft loss was also noted in a Swiss study involving 2158 solid‐organ transplant recipients with CDI (HR 2.24, 95% CI 1.15–4.37; *p* = .02).^34^ The underlying mechanism for transplant rejection after CDI is not clearly understood. Alterations in the gut microbiome may play a role in the rejection process; however, more research is needed to clarify this pathway.

It is well understood that patients with autoimmune liver disease (PSC, PBC, and AIH) have high disease recurrence post‐LT.^35^, ^36^, ^37^, ^38^, ^39^ Due to the risk of recurrence, patients with the autoimmune liver disease typically require more immunosuppression post‐LT than patients with non‐autoimmune liver disease. Interestingly, despite the increased immunosuppression, we did not find a statistically significant difference in the rates of CDI post‐LT between autoimmune and non‐autoimmune liver disease. It is unclear why increased immunosuppression does not result in increased rates of CDI. Perhaps the increased risk of CDI is only correlated to an immunocompromised state rather than the level of immunosuppression. More studies are needed to better understand the role of immunosuppression in the gastrointestinal tract and its effects on the development of CDI.

The limitations of this study are the inherent deficiencies of any retrospective analysis. Due to the retrospective nature of the database, time‐dependent analysis is not possible, especially whether CDI predisposes to rejection or LT rejection predisposes to CDI. Therefore, only measures of association were reported in the present study.

In conclusion, CDI in the LT population remains a concern due to increased mortality and healthcare utilization. Given the multiple risk factors for CDI among LT recipients, increased efforts must be taken during the pre‐ and post‐transplant period to educate patients on preventative measures to decrease CDI. As community‐acquired CDI trends toward being the predominant form of CDI, education on hygiene must be provided to family members and friends of LT recipients. Discharge instructions on index LT admission must review hand hygiene and provide education on early reporting of symptoms such as diarrhea and abdominal pain. Emphasis on improving patient education and a better understanding of treatment options such as FMT may help improve future outcomes for CDI in the LT population.

## AUTHOR CONTRIBUTIONS

Hassam Ali: conceptualization, writing‐review and editing, supervision, and project administration. Pratik Patel: investigation, writing‐original draft, visualization, proof reading. Rahul Pamarthy: investigation, resources, writing‐original draft, and visualization. Karina Fatakhova: investigation, resources, writing‐original draft, proof reading. Nicole Leigh Bolick: investigation, resources, writing‐original draft, and proof reading. Sanjaya Kumar Satapathy: investigation, resources, writing‐original draft, and proof reading.

## CONFLICTS OF INTEREST

The authors certify that they have no affiliations with or involvement in any organization or entity with any financial interest. The authors declare that they have no conflicts of interest.

## FUNDING INFORMATION

This research did not receive any specific grant from funding agencies in the public, commercial, or not‐for‐profit sectors.

## ETHICS STATEMENT

NIS contains de‐identified third‐party data. Therefore, it was deemed exempt from the institutional review board. NIS also does not include patient identifiers; therefore, patient consent was waived.

## PATIENT CONSENT STATEMENT

NIS also does not include patient identifiers; therefore, patient consent was waived.

## Supporting information

Table S1 List of ICD‐9/10 codes utilized in the present study
Table S2 Comparative analysis of characteristics for liver transplant hospitalizations with and without *Clostridium difficile* infection
Table S3 Trends of *Clostridium difficile* infection among the liver transplant population (*N*% as total number and proportion of liver transplant admissions with *C. difficile* infection out of for all liver transplant hospitalizations)

Visual Abstract

## Data Availability

The datasets generated during and analyzed during the current study are available at https://www.hcup‐us.ahrq.gov.
