# Supplementary material for: Comparative analysis and trends in liver transplant hospitalizations with Clostridium difficile infections: A 10‐year national cross‐sectional study
Source: Transpl Infect Dis. 2022 Nov 14;24(6):e13985. doi: 10.1111/tid.13985 (PMC10078594; doi:10.1111/tid.13985)
Supplement: Supplementary file 1 — Table S1 List of ICD‐9/10 codes utilized in the present study Table S2 Comparative analysis of characteristics for liver transplant hospitalizations with and without Clostridium difficile infection Table S3 Trends of Clostridium difficile infection among the liver transplant population (N% as total number and proportion of liver transplant admissions with C. difficile infection out of for all liver transplant hospitalizations) [file TID-24-0-s001.docx]

Supplementary Table 1: List of ICD-9/10 codes utilized in the present study

| Variables | ICD-9 codes | ICD-10 codes |
| --- | --- | --- |
| Liver transplant | V427, 5051, 5059 | Z944, 0FY00Z0, 0FY00Z1, 0FY00Z2 |
| Clostridium difficile infection | 00845 | A047, A0471, A0472 |
| Autoimmune etiologies of liver transplant | 5716, 57142 | K743, K8301, k754 |
| Liver transplant rejection | 99682 | T8640, T8641, T8642 |
| Inflammatory bowel disease | 5560, 5561, 5562, 5563, 5565, 5566,  5568, 5569, 5550, 5551, 5552, 5559 | K5100, K51011, K51012, K51013, K51014, K51015, K51016, K51017, K51018, K51019, K5120, K51211, K51212, K51213, K51214, K51218, K51219, K5130, K51311, K51312, K51313, K51314, K51318, K51319, K5140, K51411, K51412, K51512, K51513, K51514, K51518, K51519, K5180, K51811, K51812, K51813, K51814, K51818, K51819, K5190, K51911, K51912, K51913, K51914, K51918, K51919, K50011, K50012, K50013, K50014, K50018, K50019, k5010, K50111, K50112, K50113, K50114, K50118, K50119, k5080, K50811, K50812, K50813, K50814, K50818, K50819, k5090, K50911, K50912, K50913, K50914, K50918, K50919, k5000 |

Supplementary Table 2: Comparative analysis of characteristics for liver transplant hospitalizations with and without Clostridium difficile infection

|  | **No Clostridium difficile infection** | **Clostridium difficile infection** | **P-value** |
| --- | --- | --- | --- |
| **Total Number of Hospitalizations** | 444659 (96.64%) | 15457 (3.36%) | P < 0.001 |
| **Mean Age**  **(years)** | 57.9  ± 0.37 | 58.2  ± 0. 43 | P = 0.28 |
| **Mean LOS (days)** | 7.5  ± 0.09 | 14.5  ± 0. 50 | P < 0.001 |
| **MIC** | 123443 ± 2669 | 200419 ± 9256 | P < 0.001 |
| **Age Group**  **(years)** | **Number and %** |  | P = 0.37 |
| 18 – 34 | 32889 (24%) | 1064 (7%) |  |
| 35 – 49 | 53801 (22%) | 1909 (13%) |  |
| 50 – 64 | 214626 (25%) | 7332 (48%) |  |
| 65 – 79 | 134277 (26%) | 4885 (32%) |  |
| ≥ 80 | 329 (<1%) | 20 (<1%) |  |
| **Gender** |  |  | P < 0.001 |
| Male | 271858 (61%) | 8662 (56%) |  |
| Female | 172801 (39%) | 6795 (44%) |  |
| **Race** |  |  | P = 0.11 |
| White | 313683 (75%) | 11201 (77%) |  |
| Black | 38369 (9%) | 1223 (8%) |  |
| Hispanic | 54445 (13%) | 1810 (12%) |  |
| Asian/Native American | 11654 (3%) | 328 (2%) |  |
| **Charlson Comorbidity**  **Index Score** |  |  | P = 0.0039 |
| 0 | 53145 (12%) | 1631 (11%) |  |
| 1 | 69722 (16%) | 2154 (14%) |  |
| 2 | 61340 (14%) | 2319 (15%) |  |
| ≥ 3 | 260583 (59%) | 9352 (61%) |  |
| **Hospital**  **Region** |  |  | P < 0.001 |
| Northeast | 86208 (19%) | 3100 (20%) |  |
| Midwest | 107883 (24%) | 4053 (26%) |  |
| South | 164140 (37%) | 4866 (31%) |  |
| West | 86558 (19%) | 3437 (22%) |  |
| **Hospital Location and Teaching Status** |  |  | P < 0.001 |
| Rural | 19282 (4%) | 376 (2%) |  |
| Urban Nonteaching | 70864 (16%) | 2093 (14%) |  |
| Urban Teaching | 352454 (80%) | 12929 (84%) |  |
| **Expected Primary**  **Payer** |  |  | P = 0.27 |
| Medicare | 237707 (55%) | 8370 (56%) |  |
| Medicaid | 48228 (11%) | 1565 (10%) |  |
| Private | 142005 (33%) | 5004 (33%) |  |
| Other | 4386 (1%) | 108 (1%) |  |
| **Median Household**  **Income (Quartile)** |  |  | P < 0.001 |
| 1^st^ (0-25^th^) | 110268 (25%) | 3328 (22%) |  |
| 2^nd^ (26^th^ -50^th^) | 111279 (26%) | 3602 (24%) |  |
| 3^rd^ (51^st^ -75^th^) | 115173 (26%) | 4210 (28%) |  |
| 4^th^ (76^th^ -100^th^) | 99486 (23%) | 3939 (26%) |  |
| **Inpatient**  **Mortality** | 9224 (2%) | 595 (4%) | P < 0.001 |
| **Liver transplant rejections** | 10495 (2%) | 485 (3%) | P = 0.0035 |
| **Inflammatory bowel disease** | 14040 (3%) | 867 (6%) | P < 0.001 |
| **Auto-immune etiology** | 15139 (3%) | 318 (3%) | P = 0.9 |

Supplementary Table 3: Trends of Clostridium difficile infection among the liver transplant population (N % as total number and proportion of liver transplant admissions with Clostridium difficile infection out of for all liver transplant hospitalizations)

| **VARIABLE** |  |  | **YEARS** |  |  |  |  |  |  |  |  | **P-VALUE** |
| --- | --- | --- | --- | --- | --- | --- | --- | --- | --- | --- | --- | --- |
|  | **2009** | **2010** | **2011** | **2012** | **2013** | **2014** | **2015** | **2016** | **2017** | **2018** | **2019** |  |
| **Total Number of Hospitalizations** | 1222 (3.3%) | 1193 (3.16%) | 1221 (3.11%) | 1220 (3.2%) | 1480 (3.75%) | 1730 (4.11%) | 1045 (3.28%) | 1600 (3.53%) | 1560 (3.22%) | 1690 (3.43%) | 1495 (2.84%) | **P = 0.050** |
| **Mean Age (years)** | 56.3  ± 0.17 | 57.7  ± 0.51 | 60.4 ± 0.83 | 57.6 ± 0.23 | 57.1 ± 0.43 | 57.9  ± 0.98 | 59.4  ± 0.88 | 58.6  ± 0.25 | 59.01 ± 0.03 | 58.3  ± 0.65 | 57.9  ± 0.59 | **P = 0.1** |
| **Adjusted Mean length of stay** | 14.3  ± 0.97 | 14.1  ± 0.65 | 14.1 ± 0.66 | 13.7 ± 0.52 | 13.9 ± 0.54 | 13.8 ± 0.54 | 14.1 ± 0.54 | 13.9 ± 0.50 | 13.9 ± 0.53 | 14.1 ± 0.53 | 14.0 ± 0.50 | **P = 0.9** |
| **Adjusted Mean inpatient cost ($)** | 150517  ± 517 | 157936 ± 12821 | 171784± 17115 | 169018  ± 11171 | 175171  ± 10397 | 174136  ± 10284 | 185696 ± 11162 | 193664  ± 1328 | 198800 ± 11729 | 211499  ± 13105 | 216179 ± 13137 | **P < 0.001** |
| **Age Groups (years)** |  |  |  |  |  |  |  |  |  |  |  | **P = 0.1** |
| 18 - 34 | 68 (6%) | 74 (6%) | 36 (3%) | 60 (5%) | 135 (9%) | 150 (9%) | 75 (7%) | 125 (8%) | 105 (7%) | 110 (7%) | 125 (8%) |  |
| 34 - 49 | 200 (16%) | 134 (12%) | 88 (7%) | 160 (13%) | 200(14%) | 170 (10%) | 115 (11%) | 185 (12%) | 175 (11%) | 250 (15%) | 230(16%) |  |
| 50 - 64 | 699 (57%) | 646 (56%) | 686 (57%) | 655 (54%) | 670 (46%) | 840 (50%) | 415 (40%) | 740 (47%) | 695 (45%) | 725 (44%) | 560(38%) |  |
| 65 - 79 | 249 (20%) | 304 (26%) | 382 (32%) | 320 (27%) | 450(31%) | 535 (32%) | 425 (41%) | 520 (33%) | 560 (36%) | 575 (35%) | 565(38%) |  |
| ≥ 80 | 5 (<1%) | 0 (0%) | 0 (0%) | 10 (1%) | 0 (0%) | 0 (0%) | 0 (0%) | 5 (0%) | 0 (0%) | 0 (0%) | 0 (0%) |  |
| **Gender** |  |  |  |  |  |  |  |  |  |  |  | **P = 0.07** |
| Male | 776 (63%) | 754 (63%) | 681 (56%) | 675 (55%) | 765 (52%) | 985 (57%) | 600 (57%) | 870 (54%) | 770 (49%) | 940 (56%) | 845 (57%) |  |
| Female | 446 (37%) | 439 (37%) | 539 (44%) | 545 (45%) | 715 (48%) | 745 (43%) | 445 (43%) | 730 (46%) | 790 (51%) | 750 (44%) | 650 (43%) |  |
| **Race** |  |  |  |  |  |  |  |  |  |  |  | **P = 0.7** |
| White | 776 (75%) | 889 (81%) | 921 (78%) | 865 (75%) | 1005 (72%) | 1315 (79%) | 700 (70%) | 1140 (78%) | 1130 (77%) | 1300 (80%) | 1160 (79%) |  |
| Black | 45 (4%) | 45 (4%) | 116 (10%) | 115 (10%) | 160 (11%) | 135 (8%) | 115 (12%) | 120 (8%) | 105 (7%) | 150 (9%) | 115 (8%) |  |
| Hispanic | 166 (16%) | 143 (13%) | 124 (11%) | 140 (12%) | 200 (14%) | 180 (11%) | 145 (15%) | 175 (12%) | 210 (14%) | 165 (10%) | 160 (11%) |  |
| Asian/Native american | 39 (4%) | 20 (2%) | 14 (1%) | 30 (3%) | 30 (2%) | 40 (2%) | 35 (4%) | 35 (2%) | 30 (2%) | 20 (1%) | 35 (2%) |  |
| **Charlson Comorbidity Index (CCI)** |  |  |  |  |  |  |  |  |  |  |  | **P < 0.001** |
| CCI=0 | 168 (14%) | 260 (22%) | 211 (17%) | 190 (16%) | 295 (20%) | 320 (18%) | 180 (17%) | 0 (0%) | 0 (0%) | 5 (<1%) | 0 (0%) |  |
| CCI=1 | 136 (11%) | 164 (14%) | 173 (14%) | 130 (11%) | 225 (15%) | 200 (12%) | 170 (16%) | 305 (19%) | 220 (14%) | 250 (15%) | 180 (12%) |  |
| CCI=2 | 273 (22%) | 207 (17%) | 178 (15%) | 270 (22%) | 260 (18%) | 300 (17%) | 165 (16%) | 195 (12%) | 105 (7%) | 220 (13%) | 145 (10%) |  |
| CCI ≥ 3 | 644 (53%) | 560 (47%) | 657 (54%) | 630 (52%) | 700 (47%) | 300 (53%) | 530 (51%) | 1100 (69%) | 1235 (79%) | 1215 (72%) | 1170 (78%) |  |
| **Hospital Region** |  |  |  |  |  |  |  |  |  |  |  | **P = 0.5** |
| Northeast | 197 (16%) | 265 (22%) | 332 (27%) | 280 (23%) | 270 (18%) | 280 (16%) | 215 (21%) | 330 (21%) | 270 (17%) | 345 (20%) | 315 (21%) |  |
| Midwest | 399 (33%) | 417 (35%) | 321 (26%) | 320 (26%) | 385 (26%) | 355 (21%) | 275 (26%) | 405 (25%) | 385 (25%) | 400 (24%) | 390 (26%) |  |
| South | 182 (15%) | 327 (27%) | 341 (28%) | 285 (23%) | 525 (35%) | 750 (43%) | 340 (33%) | 545 (34%) | 505 (32%) | 605 (36%) | 460 (31%) |  |
| West | 443 (36%) | 182 (15%) | 226 (19%) | 335 (27%) | 300 (20%) | 345 (20%) | 215 (21%) | 320 (20%) | 400 (26%) | 340 (20%) | 330 (22%) |  |
| **Hospital Location and Teaching Status** |  |  |  |  |  |  |  |  |  |  |  | **P = 0.011** |
| Rural | 17 (1%) | 39 (3%) | 24 (2%) | 45 (4%) | 35 (2%) | 35 (2%) | 30 (3%) | 50 (3%) | 35 (2%) | 25(1%) | 40 (3%) |  |
| Urban Nonteaching | 199 (17%) | 250 (21%) | 258 (21%) | 210 (17%) | 260 (18%) | 200 (12%) | 120 (11%) | 150 (9%) | 190 (12%) | 145 (9%) | 110 (7%) |  |
| Urban Teaching | 970 (82%) | 898 (76%) | 921 (77%) | 965 (79%) | 1185 (80%) | 1495 (86%) | 895 (86%) | 1400 (87%) | 1335 (86%) | 1520 (90%) | 1345 (90%) |  |
| **Primary Payer** |  |  |  |  |  |  |  |  |  |  |  | **P = 0.62** |
| Medicare | 578 (50%) | 575 (49%) | 680 (58%) | 635 (54%) | 775 (53%) | 1010 (60%) | 630 (62%) | 935 (59%) | 880 (57%) | 860 (52%) | 810 (56%) |  |
| Medicaid | 157 (14%) | 133 (11%) | 108 (9%) | 155 (13%) | 165 (11%) | 140 (8%) | 85 (8%) | 165 (10%) | 150 (10%) | 175 (11%) | 130 (9%) |  |
| Private | 421 (36%) | 444 (38%) | 378 (32%) | 395 (33%) | 510 (35%) | 515 (31%) | 295 (29%) | 470 (30%) | 480 (31%) | 595 (36%) | 500 (34%) |  |
| Other | 9 (1%) | 10 (1%) | 4 (<1%) | 0 (0%) | 0 (0%) | 20 (1%) | 10 (1%) | 5 (0%) | 25 (2%) | 10 (1%) | 15 (1%) |  |
| **Median Household Income** |  |  |  |  |  |  |  |  |  |  |  | **P = 0.90** |
| 1^st^ (0-25^th^) | 242 (21%) | 179 (16%) | 307 (26%) | 265 (22%) | 370 (26%) | 370 (22%) | 260 (25%) | 330 (21%) | 345 (27%) | 350 (21%) | 310 (21%) |  |
| 2^nd^ (26^th^ -50^th^) | 310 (26%) | 244 (21%) | 267 (22%) | 250 (21%) | 315 (22%) | 430 (25%) | 210 (20%) | 385 (25%) | 405 (27%) | 400 (24%) | 385 (26%) |  |
| 3^rd^ (51^st^ -75^th^) | 312 (27%) | 343 (30%) | 309 (26%) | 355 (30%) | 365 (25%) | 480 (28%) | 305 (30%) | 470 (30%) | 370 (24%) | 470 (28%) | 430 (29%) |  |
| 4^th^ (76^th^ -100^th^) | 307 (26%) | 376 (33%) | 311 (26%) | 330 (27%) | 400 (28%) | 415 (24%) | 255 (25%) | 370 (24%) | 395 (26%) | 445 (27%) | 335 (23%) |  |
| **Liver rejection** | 62 (5%) | 54 (5%) | 53 (4%) | 25 (2%) | 50 (3%) | 90 (5%) | 40 (4%) | 25 (2%) | 45 (3%) | 10 (1%) | 30 (2%) | **P = 0.0048** |
| **Died** | 69 (6%) | 53 (4%) | 67 (6%) | 45 (4%) | 70 (5%) | 45 (3%) | 45 (4%) | 60 (4%) | 65 (4%) | 30 (2%) | 45 (3%) | **P = 0.3** |
