# Supplementary material for: Comparative analysis and trends in liver transplant hospitalizations with Clostridium difficile infections: A 10‐year national cross‐sectional study
Source: Transpl Infect Dis. 2022 Nov 14;24(6):e13985. doi: 10.1111/tid.13985 (PMC10078594; doi:10.1111/tid.13985)
Supplement: Supplementary file 2 — Visual Abstract [file TID-24-0-s002.pptx]

## Slide 1
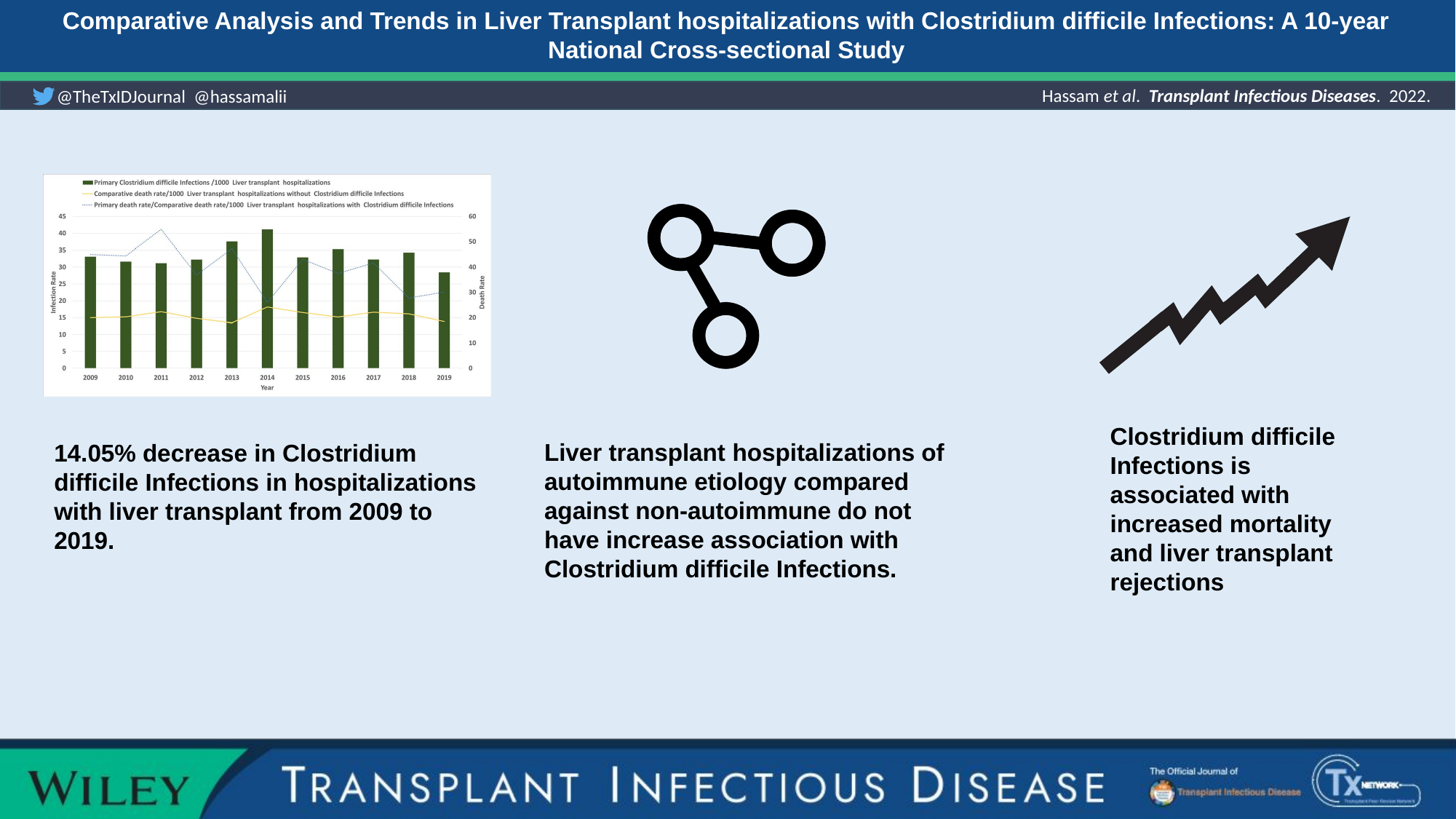

Comparative Analysis and Trends in Liver Transplant hospitalizations with Clostridium difficile Infections: A 10-year National Cross-sectional Study
Hassam et al. Transplant Infectious Diseases. 2022.
 @TheTxIDJournal @hassamalii
Clostridium difficile Infections is associated with increased mortality and liver transplant rejections
Liver transplant hospitalizations of autoimmune etiology compared against non-autoimmune do not have increase association with Clostridium difficile Infections.
14.05% decrease in Clostridium difficile Infections in hospitalizations with liver transplant from 2009 to 2019.
